# Smoking status and SARS-CoV-2 infection severity among Lebanese adults: a cross-sectional study

**DOI:** 10.1186/s12879-022-07728-1

**Published:** 2022-09-24

**Authors:** Abbas Hoballah, Rana El Haidari, Rima Badran, Ali Jaber, Samir Mansour, Linda Abou-Abbas

**Affiliations:** 1 General Director of Islamic Health Society, Baabda, Lebanon; 2 Department of Research, Islamic Health Society, Baabda, Lebanon; 3 Department of Psychological Health, Islamic Health Society, Baabda, Lebanon; 4 Department of Informatics, Islamic Health Society, Baabda, Lebanon; 5 Neuroscience Research Center, Faculty of Medical Sciences, Lebanese University, Beirut, Lebanon; 6 Epidemiological Surveillance Program, Ministry of Public Health, Beirut, Lebanon

**Keywords:** Smoking status, SARS-CoV-2 infection severity, Lebanese adults, Cross-sectional study

## Abstract

**Background:**

A paradoxical hypothesis about the effect of smoking on patients infected with severe acute respiratory syndrom 2 (SARS-CoV-2) infection still exists. Furthermore, gender-discrepancy in the impact of smoking on COVID-19 severity was given little attention. Thus, the aims of the present study were to evaluate the prevalence of smoking and the COVID-19 infection severity in a sample of adult patients diagnosed with COVID-19 and to explore the relationship between smoking status and SARS-CoV-2 infection severity in the overall sample and stratified by gender.

**Methods:**

A retrospective analytical study was conducted on patients diagnosed with COVID-19 cases between December, 2020 and April, 2021 from three leading laboratories in Lebanon. Sociodemographic characteristics, smoking status and clinical symptoms were collected. Multinomial logistic regression analysis was used to explore the relationship between smoking status and SARS-CoV-2 infection severity.

**Results:**

A total of 901 confirmed COVID-19 cases participated in the study, 50.8% were females. The mean age of patients was 38.4 years (SD = 15.3). Of the total sample, 521(57.8%) were current smokers. Regarding infection severity, 14.8% were asymptomatic, 69.9% had mild symptoms, while 15.3% had severe infection. In the overall sample, smoking status, smoking types and dose–response were not significantly associated with infection severity. Upon stratifying the entire sample by gender, no association was found between all the considered variables with infection severity among females. However, a significant association was found among male with mild infection compared to their asymptomatic counterparts (OR = 1.78 95% CI (1.01–3.13)). Waterpipe smoking was found to be associated with infection severity among male with mild infection (OR 2.64 (95% CI 1.32–5.27)) and severe infection 2.79, 95% CI (1.19–6.53) compared to their asymptomatic counterparts.

**Conclusion:**

Our fundings highlight sex differences in the association between tobacco smoking and COVID-19 severity. Current tobacco smoking was not associated with SARS-CoV-2 infection severity among female patients, however, tobacco smoking, particularly waterpipe, was found to be associated with infection severity among male. Thus, the battle against smoking should continue by assisting smokers to successfully and permanently quit.

## Introduction

The severe acute respiratory syndrome coronavirus 2 (SARS-CoV-2), the causative agent of coronavirus disease (COVID-19), has rapidly swept the globe, producing devastating threats on global public health and economy [1]. SARS-CoV-2 holds a higher aggressive and contagious capacity than any prior human coronavirus [2]. Tremendous efforts have been made to better understand transmission, mechanisms and pathophysiology, as well as developing new diagnostic, preventative and therapeutic measures. Studies have identified that the cell surface molecule angiotensin-converting enzyme 2 (ACE2) serves as the prominent host receptor and mediates the process of SARS-CoV-2 infection in human cells [3–5]. In particular, the SARS-CoV-2 spike (S) protein binds to the cellular ACE2-mediates the subsequent fusion between viral envelope and host cell membrane through receptor-mediated endocytosis, thereby allowing viral entry into host cells [6]. Globally, several strategies were adopted to fight SARS-CoV-2 induced alveolar damages and reduce the symptoms’ severity [7].

As this new virus spreads, more questions about COVID-19 disease severity risk factors arise. Observational studies have consistently identified several risk factors including older age, male sex and comorbidities such as cancer, hypertension, diabetes and respiratory diseases [8–10]. However, studies evaluating the impact of smoking on COVID-19 vulnerability have shown contradictory results. Whilst a protective effect existed in research conducted early during the pandemic with lower smoking prevalence among COVID-19 patients compared to the general population, a positive association was reported in other studies suggesting that smoking would worsen the SARS‐CoV‐2 prognosis including hospitalization in an intensive care unit, the need for mechanical ventilation, or mortality [11–14]. In addition, some studies revealed no association between smoking and SARS-CoV-2 infection [15, 16].

Along with the conflicting observational evidence that a "smoker's paradox" exists in COVID-19, several mechanisms have been proposed [17–20]. Authors postulate that nicotine would protect against COVID-19 through the SARS-CoV-2 penetration and propagation inhibition [21, 22]. Within the respiratory tract and central nervous system, nicotine is thought to compete with SARS-CoV-2 for the nicotinic acetylcholine receptor binding site that works as a co-receptor for viral cell entrance resulting in a decrease in accessible viral adhesion sites [23]. To support this hypothesis, several studies were initiated to elucidate the relationship between nicotine and SARS-CoV-2 infection severity [16, 24, 25]. Conversely, a hypothetical relationship between smoking and severe COVID-19 symptoms can be made as smoking was consistently found to be associated with increased morbidity and mortality in a variety of respiratory infections due to its immune response's suppressive effect [17, 26]. Gene expression and subsequent receptor levels are elevated in the airway and oral epithelium of current smokers, consequently putting smokers at higher risk of contracting SARS-CoV-2 [27, 28]. It is worth noting that the majority of the published studies that falls under the scope of smoking and SARS-CoV-2 severity were conducted among hospitalized COVID-19 patients [29]. The latter represents only a minority among SARS-CoV-2 confirmed population. However, asymptomatic and clinically mild infections that do not necessitate hospitalization are more likely to occur. Thus, it would be interesting to evaluate the prevalence of smoking and to clarify the association between smoking and COVID-19 severity in a sample of COVID-19 patients that present the full spectrum of disease severity ranging from asymptomatic to severe disease level.

Lebanon, a small developing country located on the eastern shore of the Mediterranean Sea, was ranked third in the world for having the most smokers per capita for both sexes [30]. The World Health Organisation estimates of 2018 revealed that the prevalence of current tobacco smoking in Lebanon is 42.6% [31]. The most recent household survey revealed a significatly higher cigarette smoking rate in males than females (48.6% and 21.5% respectively) and a high prevalence of waterpipe smoking with 32.7% and 46.2% males and females respectively [32]. Therefore, assessing the association between smoking and COVID-19 severity in Lebanon is greatly representative and instructive. Thus, the aims of the present study were to evaluate the prevalence of smoking and the COVID-19 infection severity in a sample of adult patients diagnosed with COVID-19. In addition, we sought to explore the relationship between smoking and SARS-CoV-2 infection severity.

## Materials and methods

### Data source

An observational retrospective analytical study was conducted on patients diagnosed with COVID-19 at the Islamic Health Society (IHS): one of the largest non-governmental organizations in Lebanon. All the electronic laboratory records of confirmed SARS-CoV-2 cases in three IHS laboratories between December, 2020 and April, 2021 were accessed. Adult patients aged 18 years and above and who tested positive for SARS-CoV-2 by the reverse-transcriptase–polymerase chain reaction testing (RT-PCR) of nasopharyngeal swab were eligible to participate in the study. Eligible patients were contacted via phone by the IHS “Corona Call Center”, and were asked to provide an oral informed consent to participate in the study. The study protocol was approved by the Islamic Health Society Research Ethics Committee (11-21,421-SC).

### Data collection

Participants were asked to provide data regarding their sociodemographic characteristics (age, gender, education), weight, height, diverse comorbidities, smoking behaviors (current smoking consumption, type of tobacco, frequency and intensity of current smoking and duration of smoking), previous SARS-CoV-2 infection (symptoms, duration, and severity), treatment, and hospitalization (admission).

### Main outcomes

Smoking status was defined as never smoked (i.e. persons who had never smoked regularly), and current smokers. To explore the dose–response effect, a variable by collapsing data on smoking status and smoking duration in years was created as follows: current smokers grouped with mild smokers (≤ 10 cigarettes/day for < 15 years), moderate smokers (≤ 10 cigarettes/day for ≥ 15 years or > 10 cigarettes/day for < 15 years), and heavy smokers (> 10 cigarettes/day for ≥ 15 years) [16]. For waterpipe smokers, participants were grouped with mild smokers (≤ 4 waterpipe /week for < 15 years), moderate smokers (≤ 4 waterpipe /week for ≥ 15 years or > 4 waterpipe /week for < 15 years), and heavy smokers (> 4 waterpipe /week for ≥ 15 years). To calculate the cumulative dosing of cigarette/waterpipe smoking, smoking duration (in years) was multiplied by the mean number of daily cigarette packs or number of times waterpipes were used weekly.

Patients with COVID-19 were classified into three levels:

- Asymptomatic infection: absence of SARS-CoV-2 symptoms;
- Mild infection: presence of at least one SARS-CoV-2 symptoms excluding pneumonia;
- Moderate/Severe infection: presence of pneumonia and/or hospitalization for COVID-19 [16].

### Sample size calculation

The sample size was calculated using the Epi Info™ tool (Center for Disease Control, Atlanta, GA, USA). Available from http://www.cdc.gov/epiinfo/. A minimal sample size of 384 was calculated assuming a priori estimated 42.6% smoking prevalence in Lebanese population [31], a confidence interval of 95%, a maximum allowable error in the prevalence of 1%, and a Lebanese population size of 4,842,000 habitants based on the latest Lebanese census data [35].

### Statistical analysis

Sociodemographic and clinical characteristics of the participants were described using the mean (standard deviation) for continuous variables, and the number (percent) for qualitative variables. Categorical variables were compared in univariate analyses (Pearson chi square test) and the means of continuous variables with the Student’s t test or the one-way analysis of variance (ANOVA). Bivariate multinomial logistic regression models (overall, split by gender) were conducted to explore the association between smoking status and dosages and SARS-CoV-2 infection severity. The infection severity variable (asymptomatic, mild and severe) was considered as dependent variable using “asymptomatic” as a reference category; smoking status (smokers versus non-smokers) and dosages variables such as smoking type (non-smokers, cigarette smokers, waterpipe smokers,and dual smokers); dose–response relationship for cigarette (non-smokers, mild, moderate, heavy) and waterpipe (non-smokers, mild, moderate, heavy) were considered as independent variables. Independent variables with p-value < 0.2 were included in the model. The covariables used for each model were age, body mass index and comorbidities (no versus yes). Associations were estimated with odds ratios (ORs) along with their corresponding 95% confidence intervals. The akaike information criterion (AIC) was computed to determine how the data fit the regression model. All tests were two-sided and statistical significance was set at p-value < 0.05. The collected data was analyzed with the Statistical Package for Social Sciences software (SPSS) version 26.

## Results

A total of 901 confirmed COVID-19 cases participated in the study of whom 50.8% were females. The mean age of patients was 38.4 years (SD = 15.3). About one third of the participants (34.9%) had a university education level. Patients with underlying disease represented 31.3% of the total. Out of 901 patients, 521(57.8%) were current smokers, out of whom 41.8% were cigarette smokers, 52.8% were waterpipe smokers and 5.4% were dual smokers. Demographic and clinical characteristics of the COVID-19 patients as per their smoking status are shown in Table 1. Smokers were more likely to be males (58.2% males vs 41.8% females) and to have a lower mean age (38.3 years) as compared to non-smokers (38.6 years). Comorbidities among smokers were higher than among non-smokers (69.1% vs 30.9%).

**Table 1:** Sociodemographic and clinical characteristics of the study participants by smoking status (N = 901)

Variables	All participants (N = 901)	Non-smoker (n = 380)	Smoker (n = 521)	p-value	Cigarette smoking (n = 218)			p-value	Waterpipe smoking (n = 275)			p-value	Cigarette and waterpippe smoking (n = 28)
					Mild (n = 19)	Moderate (n = 101)	Heavy (n = 93)		Mild (n = 58)	Moderate (n = 196)	Heavy (n = 21)		
Age, years mean (SD)	38.4 (15.3)	38.6 (15.7)	38.3 (15.1)	0.73	32.6 (13.8)	37.8 (15.5)	53.1 (14.2)	< 0.0001	33.8 (10.6)	33.4 (12.1)	39.7 (8.1)	0.06	33.0 (12.4)
Gender													
Male	458 (49.2)	155 (40.8)	303 (58.2)	< 0.0001	11 (7.6)	74 (51.0)	60 (41.4)	0.25	26 (44.8)	94 (48.0)	14 (66.7)	0.20	20 (71.4)
Female	443 (50.8)	225 (59.2)	218 (41.8)		8 (11.8)	27 (39.7)	33 (48.5)		32 (55.2)	102 (52.0)	7 (33.3)		8 (28.6)
Education													
Primary school	212 (23.5)	78 (36.8)	134 (63.2)	0.01	3 (4.1)	29 (39.7)	41 (56.2)	0.05	11 (23.4)	9 (19.1)	27 (57.4)	0.27	6 (21.4)
Middle or high school	199 (22.1)	68 (34.2)	131 (65.8)		6 (10.5)	32 (56.1)	19.93(3.3)		38 (23.2)	49 (29.9)	77 (47.0)		9 (32.1)
University or postgraduate degree	314 (34.9)	146 (46.5)	168 (53.5)		7 (13.7)	23 (45.1)	21 (41.2)		5 (33.3)	6 (40.0)	4 (26.7)		8 (28.6)
BMI, (kg/m^2^) mean (SD)	26.6 (5.1)	26.3 (5.1)	26.8 (5.2)	0.14	24.6 (5.9)	26.2 (4.10)	28.4 (5.3)	0.001	26.1 (5.4)	26.7 (5.6)	28.3 (3.6)	0.29	25.9 (3.9)
Comorbidities (yes)	282 (31.3)	120 (42.6)	162 (57.4)	0.88	5 (5.6)	27 (30.3)	57 (64.0)	< 0.0001	14 (22.2)	41 (65.1)	8 (12.7)	0.20	5 (17.9)

Comparisons of demographic and clinical information between asymptomatic patients and those with mild or severe disease are shown in Table 2. Infection severity was associated with age, gender, educational level and comorbidities (p-value˂0.05). The highest mean age was found among patients who had a severe infection compared to their asymtomatic counterparts or patients with mild infection (47.0 years old for severe cases versus 36.9 years for mild and asymptomatic patients (p-value < 0.0001)). Men had more severe infection than women (17.9% versus 12.6%; p-value = 0.03). Participants with primary school were more likely to suffer from severe infection compared to their counterparts with high education level (24.5% versus 9.5% and 12.7% for Middle or high school and university educational level respectively; p < 0.0001). Results also showed that COVID-19 cases with comorbidities had high proportion of severe infection compared to their asymtomatic counterparts or patients with mild infection (49.3% versus 28.9% and 24.1% for mild and asymptomatic patients respectively; p < 0.0001) (Table 2).

**Table 2:** Sociodemographic and clinical characteristics of the study participants by infection severity

Variables	All participants (n = 901)	Infection severity*			p-value
		Asymptomatic infection (n = 133)	Mild (n = 630)	Moderate/severe (n = 138)	
Age, years mean (SD)	38.4 (15.3)	36.9 (14.7)	36.9 (14.7)	47.0 (15.8)	< 0.0001^†^
Gender n (%)					
Male	458 (49.2)	73 (15.3)	303 (66.2)	82 (17.9)	0.03^††^
Female	443 (50.8)	60 (13.5)	327 (73.8)	56 (12.6)	
Education n (%)					
Primary school	212 (23.5)	29 (13.7)	131 (61.8)	52 (24.5)	< 0.0001^††^
Middle or high school	199 (22.1)	37 (18.6)	143 (71.9)	19 (9.5)	
University	314 (34.9)	44 (14.0)	230 (73.2)	40 (12.7)	
BMI, (kg/m^2^) mean (SD)	26.6 (5.1)	26.4 (4.5)	26.5 (5.1)	27.5 (5.6)	0.12^†^
Comorbidities n (%)	282 (31.3)	32 (24.1)	182 (28.9)	68 (49.3)	< 0.0001^††^

^†^ANOVA test; ^††^Chi-square test, *Infection severity: asymptomatic infection: without SARS-CoV-2 symptoms; mild infection: at least one SARS-CoV-2 symptoms excluding pneumonia; severe infection: presence of 3 or more SARS-CoV-2 symptoms with pneumonia and/or hospitalisation for SARS-CoV-2

Table 3 shows the COVID-19 clinical characteristics and smoking status of the study participants. Of the total patients, 14.8% were asymptomatic, 69.9% had mild symptoms, while 15.3% had severe infection. Among the symptomatic patients, the most common symptoms were headache (59.4%), muscle or bone pain (53.5%), fever (46.9%) and cough (44.2%). Results showed that no significant association between infection severity, self reported SARS-CoV-2 symptoms, hospitalisation and cigarette smoking (p-value ˃0.05). Moreover, no significant associations between infection severity, hospitalisation and waterpipe smoking were found. Regarding self-reported symptoms, on the other hand, patients who smoked had olfactory and taste disorders approximately double than non-smokers (66.9% versus 33.1%) (p-value = 0.02). Furthermore, non-smokers were more fatigue than smokers (45.8% versus 54.2%, P-value = 0.01). Patients who smoked cigarette moderately and heavily had significantly higher percentage of cough (41.3% and 53.8% for moderate and heavy smokers, respectively) compared to their mild counterparts (5.0%) (p-value = 0.04) (Table 3).

**Table 3:** COVID-19 Clinical characteristics of the study participants by smoking status (n = 901)

Variables	All participants (n = 901)	Never smoked (n = 380)	Smoker (n = 521)	p-value	Cigarette smokers (n = 218)			p-value	Waterpipe smokers (n = 275)			p-value	Cigarette and Waterpippe smoking (n = 28)	p-value
					Mild (n = 19)	Moderate (n = 101)	Heavy (n = 93)		Mild (n = 58)	Moderate (n = 196)	Heavy (n = 21)			
Infection severity * n (%)														
Asymptomatic infection	133 (14.8)	63 (47.4)	70 (52.6)		1 (3.1)	17 (53.1)	14 (43.8)		6 (10.3)	28 (14.3)	1 (4.8)		2 (7.1)	0.17
Mild	630 (69.9)	256 (40.6)	374 (59.4)	0.31	16 (10.8)	70 (47.3)	62 (41.9)	0.55	42 (72.4)	141 (71.9)	16 (76.2)	0.68	24 (85.8)	
Severe	138 (15.3)	61 (44.2)	77 (55.8)		2 (6.1)	14 (42.4)	17 (51.5)		10 (17.2)	27 (13.8)	4 (19.0)		2 (7.1)	
Self-reported symptoms n (%)														
Headache	535 (59.4)	215 (40.2)	320 (59.8)	0.15	12 (9.4)	65 (50.8)	51 (39.8)	0.37	42 (72.4)	116 (59.2)	10 (47.6)	0.08	20 (71.4)	0.24
Muscle or bone pain	482 (53.5)	205 (42.4)	278 (57.6)	0.89	10 (9.3)	49 (45.8)	48 (44.9)	0.89	36 (62.1)	100 (51.0)	14 (66.7)	0.17	18 (64.3)	0.33
Fever	423 (46.9)	188 (44.4)	235 (55.6)	0.20	10 (11.6)	40 (46.5)	36 (41.9)	0.53	29 (50.0)	89 (45.4)	11 (52.4)	0.75	16 (57.1)	0.33
Cough	398 (44.2)	182 (45.7)	216 (54.3)	0.05	4 (5.0)	33 (41.3)	43 (53.8)	**0.04**	27 (46.6)	81 (41.3)	12 (57.1)	0.35	14 (50.0)	0.56
Dyspnea	290 (32.2)	123 (42.4)	167 (57.6)	0.94	8 (11.1)	28 (38.9)	36 (50.0)	0.21	15 (25.9)	60 (30.6)	8 (38.1)	0.58	11 (39.3)	0.53
Gastrointestinal distrubances	169 (18.8)	78 (46.2)	91 (53.8)	0.26	6 (16.7)	14 (38.9)	16 (44.4)	0.15	11 (19.0)	36 (18.4)	4 (19.0)	1.00	3 (10.7)	0.33
Olfactory and taste disorders	133 (14.8)	44 (33.1)	89 (66.9)	**0.02**	1 (3.6)	16 (57.1)	11 (39.3)	0.39	13 (22.4)	36 (18.4)	4 (19.0)	0.76	8 (28.6)	0.06
Fatigue	96 (10.7)	52 (54.2)	44 (45.8)	**0.01**	2 (13.3)	7 (46.7)	6 (40.0)	0.84	6 (10.3)	18 (9.2)	4 (19.0)	0.39	1 (3.6)	0.34
Sore throat or rhinorrhea	32 (3.6)	17 (53.1)	15 (46.9)	0.20	1 (16.7)	3 (50.0)	2 (33.3)	1.00	2 (3.4)	7 (3.6)	0 (0.0)	0.77	0 (0.0)	0.61
Pneumonia	21 (2.3)	8 (38.1)	13 (61.9)	0.82	0 (0.0)	2 (66.7)	1 (33.3)	1.00	2 (3.4)	5 (2.6)	1 (4.8)	1.00	1 (3.6)	1.00
Conjuctivitis	8 (0.9)	6 (75.0)	2 (25.0)	0.07	1 (100)	0 (0.0)	0 (0.0)	0.08	0 (0.0)	0 (0.0)	0 (0.0)	**–**	1 (3.6)	0.22
Heart palpitations	4 (0.4)	2 (50.0)	2 (50.0)	1.00	0 (0.0)	0 (0.0)	0 (0.0)	–	0 (0.0)	1 (0.5)	1 (4.8)	0.19	0 (0.0)	1.00
Shortness of breath	3 (0.3)	0 (0.0)	3 (100.0)	0.26	0 (0.0)	0 (0.0)	1 (0.0)	0.52	0 (0.0)	2 (1.0)	0 (0.0)	1.00	0 (0.0)	1.00
Hosptalisation (yes) n (%)	128 (14.2)	58 (45.3)	70 (54.7)	0.33	2 (6.3)	14 (43.8)	16 (50.0)	0.68	9 (16.4)	24 (12.7)	4 (19.0)	0.63	1 (3.6)	0.10

*n* frequency, % percentage

P-value ˂0.05 is considered significant

*Infection severity: asymptomatic infection: without SARS-CoV-2 symptoms; mild infection: at least one SARS-CoV-2 symptoms excluding pneumonia; severe infection: presence of 3 or more SARS-CoV-2 symptoms with pneumonia and/or hospitalisation for SARS-CoV-2

Table 4 shows adjusted ORs with 95% CI from multinomial logistic regression, with the severity groups as the dependent variable (asymptomatic group as reference), and smoking status and dosages as independent variables. In the overall sample, smoking status, smoking types and dose–response were found no significant at 5% level of significance. Upon stratifying the entire sample by gender, no association was found between all the considered variables with infection severity among females. However, a significant association was found among male with mild infection compared to their asymptomatic counterparts (OR = 1.78 95% CI (1.01–3.13). Waterpipe smoking was found to be associated with infection severity among male with mild infection (OR 2.64 (95% CI 1.32 and 5.27) and severe infection 2.79, 95% CI (1.19–6.53) compared to their asymptomatic counterparts. Regarding waterpipe dose response relationship, moderate dose consumption was associated with infection severity among male with severe infection compared to their asymptomatic counterparts (OR = 2.48, 95% CI 1.06–5.79).

**Table 4:** Adjusted odds ratio (OR) with 95% confidence intervals (CIs) from bivariate multinomial logistic regression of COVID-19 severity groups and smoking status and dosages (N = 901)

	All (N = 901)		Males		Females	
Variables	Mild versus asymptomatic OR^a^ (95% CI)	Severe versus asymptomatic OR^a^ (95% CI)	Mild versus asymptomatic OR^b^ (95% CI)	Severe versus asymptomatic OR^b^ (95% CI)	Mild versus asymptomatic OR^b^(95% CI)	Severe versus asymptomatic OR^b^ (95% CI)
Smoking status	1.23 (0.81–1.85)	1.07 (0.62–1.83)	**1.78 (1.01–3.13)**	1.44 (0.70–2.96)	0.84 (0.46–1.53)	0.77 (0.34–1.73)
AIC	1249		685		571	
Smoking type						
Non-smoker	1.00	1.00	1.00	1.00	1.00	1.00
Cigarette smoker	1.11 (0.69–1.79)	0.80 (0.43–1.48)	1.65 (0.90–3.03)	1.16 (0.53–6.53)	0.92 (0.39–2.13)	0.50(0.16–1.51)
Waterpipe smoker	1.42 (0.90–2.24)	1.59 (0.88–2.87)	**2.64 (1.32–5.27)**	**2.79 (1.19–6.53)**	0.86 (0.46–1.60)	0.91(0.39–2.14)
Dual smokers	3.03 (0.70.13.2)	1.38 (0.18–10.30)	3.20 (0.69–14.80)	1.75 (0.22–13.8)	–	–
AIC	1249		685		571	
Dose–response relationship						
Cigarette smoking						
Non-smoker	1.00	1.00	1.00	1.00	1.00	1.00
Mild	0.69 (0.17–2.83)	0.80 (0.13–4.92)	0.98 (0.17–5.47)	0.65 (0.06–6.20)	0.35 (0.02–4.57)	2.64(0.13–51.95)
Moderate	0.94 (0.44–2.00)	0.72 (0.26–1.99)	1.66 (0.60–4.56)	1.02 (0.25–4.13)	0.43 (0.13–1.42)	0.46(0.10–2.15)
Heavy	2.01 (0.56–7.18)	0.83 (0.12–5.71)	1.55 (0.36–6.69)	1.21 (0.14–9.98)	–	1.20(0.01–0.00)
AIC	1248		685		569	
Waterpipe smoking						
Non-smoker	1.00	1.00	1.00	1.00	1.00	1.00
Mild	2.11(0.24–18.24)	1.94(0.16–22.46)	–	–	0.58(0.21–1.58)	1.26(0.35–4.46)
Moderate	0.86(0.45–1.63)	0.77(0.30–1.95)	1.93 (0.97–3.84)	**2.48 (1.06–5.79)**	0.96(0.49–1.89)	0.63(0.23–1.75)
Heavy	3.15(0.89–11.12)	2.61(0.57–11.83)	4.61 (0.58–36.85)	3.07 (0.26–36.10)	–	–
AIC	1249		685		571	

Significant results are presented on bold

Cigarette smoking: Mild smokers: ≤ 10 cigarettes/day for ˂15 years, moderate smokers: ≥ 10 cigarettes/day for ˂15 years or ˃10 cigarettes/day for ˂15 years, and heavy smokers: > 10 cigarettes/day for ≥ 15 years. Waterpipe smoking: Mild smokers (≤ 4 waterpipe /week for < 15 years), moderate smokers (≤ 4 waterpipe /week for ≥ 15 years or > 4 waterpipe /week for < 15 years), and heavy smokers (> 4 waterpipe /week for ≥ 15 years)

*AIC* the akaike information criterion, *CI* confidence interval, *OR* odds ratio

^a^Models adjusted for age, gender, BMI, and comorbidities

^b^Models adjusted for age, BMI, and comorbidities

## Discussion

The aim of the present study was to evaluate the prevalence of smoking in a sample of adult patients diagnosed with COVID-19 and to explore the relationship between smoking and SARS-CoV-2 infection severity. Findings showed a high tobacco smoking prevalence among the 901 SARS-CoV-2 confirmed cases. Regarding infection severity, 14.8% were asymptomatic, 69.9% had mild symptoms, while 15.3% had severe infection. In the overall sample, smoking status, smoking types and dose–response were not significantly associated with infection severity. Upon stratifying the entire sample by gender, no association was found between all the considered variables with infection severity among females. However, a significant association was found among male with mild infection compared to their asymptomatic counterparts. Waterpipe smoking was found to be associated with infection severity among male with mild infection and severe infection compared to their asymptomatic counterparts.

In our sample, smoking prevalence was 57.8% exceeding the WHO prevalence estimates of current tobacco smoking in 2018 (42.6%) [31], as well as the prevalence of tobacco smoking reported among the hospitalized COVID-19 patients of Khalil et al. study (42.3%) [33]. Results also revealed a high prevalence of tobacco smoking among male participants (58.2%) compared to their females counterparts (41.8%) which also exceeded the WHO prevalence estimates of current tobacco (49.2% and 35.9% males and females respectively) [31]. In our sample, only 14.8% of COVID-19 cases were asymptomatic. A possible explanation of the high tobacco smoking prevalence and the low prevalence of asymptomatic cases is that PCR testing is likely to be limited to vulnerable symptomatic subgroups, with the potential for these groups to include an overrepresentation of current tobacco smokers. Moreover, due to the increased prevalence of COVID-19-related symptoms, such as cough, increased sputum production, or altered taste or smell, current tobacco smokers may be more likely to present for testing, raising the denominator in comparisons to non-smokers and potentially inflating the prevalence of SARS-CoV-2 infection among current smoker. Thus, the estimation of the SARS-CoV-2 infection positivity rates from random samples are more useful.

Findings showed that male patients present severe infection more than female. Based on previous studies, evidence suggests that male patients are the most susceptible to SARS-CoV-2 infection, which is supported by our data [36–38]. In a large global meta-analysis of 107 COVID-19 reports, Peckham et al. demonstrate that male sex is associated with a significantly increased risk of intensive treatment unit admission and higher odds of death compared to females [36]. Capuano et al. established that this sex difference may be related to several factors such as activity of the immune system and its modulation by sex hormones, coagulation pattern, and preexisting cardiovascular diseases as well as effects deriving from smoking and drinking habits [37].

No significant association was found between the smoking status and infection severity in the overall sample. This results was consistent with other studies [15, 16]. However, upon stratifying the sample by gender, multinomial logistic regression models revealed a significant association among male with mild infection compared to their asymptomatic counterparts (OR = 1.78, 95% CI (1.01–3.13)), a recent study conducted in Lebanon among 743 hospitalized COVID-19 patients revealed a high smoking prevalence (42.3%) combined with worse prognosis as well as a higher mortality rate in smoking patients [33]. A gender-discrepancy in the association of smoking on COVID-19 mortality rates was also reported. Remarkably, current smoking status was associated with higher vulnerability to death among COVID-19 hospitalized male patients, while it does not affect ICU admission or survival outcomes among hospitalized COVID-19 female patients as compared to their non-smoker counterpart [33]. More research on the potential gender-discrepancy in the effect of smoking on COVID-19 severity while accounting for smoking dosages are still needed.

Since SARS-CoV-2 infection primarily targets the lungs, smoking-related lung disorders overlap with COVID-19 respiratory comorbidities such as chronic bronchitis, emphysema, and chronic obstructive pulmonary disease (COPD) [42]. A recent study revealed that the activation of the ACE-2 in mice lungs following e-cigarette vapor exposure is gender-specific [43]. Male mice had a more pronounced nicotine-dependent increase in lung ACE-2 expression than female mice, which can influence COVID-19 severity [43]. Thus, the observed significant repercussions of smoking among men in our study could be explained by the fact that this group had a higher risk of developing lung problems as a result of tobacco smoking. Furthermore, future research most be highlight the different factors that can threaten people to infected with COVID-19 infection such as the social practices of waterpipe use. Interestingly, our results are in line with recent study who revealed that current smoking status reduces survival rate in male patients but it does not affect survival outcomes among hospitalized female patients [33]. Particularly, waterpipe smoking among males was associated with mild infection (OR 2.64, 95% CI (1.32–5.27)) and severe infection (OR 2.79, 95% CI (1.19–6.53) compared to their asymptomatic counterparts. Regarding waterpipe dose response relationship, moderate dose consumption was associated with infection severity among male with severe infection compared to their asymptomatic counterparts (OR = 2.48, 95% CI 1.06–5.79). However, a closer look to the adjusted odds ration revealed an association that did not reach statistical significance due to the low sample size in subcategories. Overall, our results suggest adding tobacco smoking particularly waterpipe as a risk factor for worse COVID-19 prognosis among males and highlight the importance of waterpipe smoking cessation.

The present study has several limitations. Due to the observational nature of the study and the cross-sectional design, we cannot infer any causal relationship between smoking habits and SARS-CoV-2 infection severity. Furthermore, smoking habits and SARS-CoV-2 symptoms were self-reported; consequently, recall bias might have led to misclassification of the exposure. Lastly, the sample with an acceptable geographical coverage reflecting the distribution of SARS-CoV-2 infection in Lebanon was not entirely representative of the Lebanese population.

## Conclusions

Our fndings highlight sex differences in the association between tobacco smoking and COVID-19 severity. Current tobacco smoking was not associated with SARS-COV-2 infection severity among female patients, however, tobacco smoking, particularly waterpipe, was found to be associated with infection severity among male. Thus, there is a growing need to support the WHO statement that ‘smokers are at higher risk of developing severe disease and death’ and the battle against smoking should continue, by assisting smokers to successfully and permanently quit [44]. Smoking cessation should be incorporated into public health campaigns especially during the SARS-CoV-2 pandemic and in the aftermath. Future studies should take into consideration social factors that affect smoking (e.g. participation in waterpipe smoking at the same time, dual smokers of cigarette and waterpipe, using several type of tobacco) in addition to comparing patients with COVID-19 to uninfected people.

## Data Availability

The datasets used and/or analysed during the current study are available from the corresponding author on reasonable request.

## Abbreviations

BMI: Body Mass Index
COVID-19: Coronavirus disease 2019
SARS-CoV-2: Severe acute respiratory syndrome coronavirus 2
CI: Confidence interval
SD: Standard deviation
RT-PCR: Reverse transcription polymerase chain reaction
